# Critical incident reporting and learning system: The black pearls

**DOI:** 10.4103/0019-5049.65348

**Published:** 2010

**Authors:** SS Harsoor

**Affiliations:** Editor, Indian Journal of Anesthesia, No. 21, 2^nd^ Cross, Kirloskar Colony, Basaveshwar Nagar, 2^nd^ Stage, Bangalore – 560 079, India. E-mail: harsoorss@hotmail.com

With the technological advances and availability of better drugs, the routine administration of anaesthesia has become quite safe in today’s clinical practice. At the same time, there is growing awareness about medication errors amongst the patients and the anaesthesiologists world over, especially when the errors cause major harm to the patient, including mortality. Such medication errors are now a global public health problem. The NHS litigation database during the period from 1995 to 2007 in the UK showed 93 litigation claims amounting to £5 million.[1] Among them, 15 claims were related to severe harm or death of patient following administration of anaesthesia. The patient safety incidents were received from critical care units to UK National Patient Safety Agency; the commonest incident (40%) group was found to be medication errors.[2] According to the available information, serious efforts have not been made in India to have any critical incident directory till this date in spite of tremendous improvement in utilisation of information technology in patient care services and medical record keeping.

The anaesthesia-related morbidity and mortality is used as an indicator of safe and quality care rendered by any institution.[3] The practice of anaesthesiology and critical care is a high risk clinical practice and hence there is a growing need for the use of critical incident reporting system. It is observed that mere identification of errors will help in a great way in minimising the errors and mitigating the problems of suffering patients. What we need is to imbibe a safety culture of learning from the past mistakes. We have to develop a culture of reporting and accepting errors and not a culture of blaming for the error.[4] Regardless of hierarchical position of the physician involved, we need to discuss all the mistakes. The famous adage of “King Makes No Mistakes” is not acceptable any more in the present days of maintaining transparency in every profession. Error detection is the fundamental necessity for further prevention. The incident reporting discloses these errors which later trigger warnings and ultimately can create a culture of safe practice. This incident reporting system may have components such as process errors which include treatment errors, communication errors or investigation errors and the other broader group – Knowledge or skill errors.[5]

There might be errors due to wrong attitude as well. The slips and lapses constitute simplest of the medication errors at one end of the spectrum, compared to the other end of deliberate violations, which are an extreme degree of medication errors. The institutions which have a tendency to blame frontline workers are more prone to such extreme degree of deliberate violations.[6] In such circumstances, it is seldom realised that errors at the sharp end can result in disastrous outcomes for those suffering patients.

Certain retroactive and proactive tools suggested for these incident reporting systems include Failure Mode, Effect and Criticality Analysis (FMECA) along with medical auditing.[7] Auditing of such incidents will help us in comparing what is done against the accepted reference standards, so that corrective steps to improve the performance will emerge from such audits. Therefore, robust error reporting ensures an anaesthetist with tremendous psychological safety and confidence to learn from past mistakes.[6]

Such critical incident reporting systems cannot be created overnight and apparently they will have initial difficulties of non-acceptance.[8] Only experience can take us forward with an expectation of learning to minimise the reported errors by the readers. One such incident reporting system already established with a precondition of its usage for improvements in overall quality of care is available for German speaking countries[5] (JFJ; www.jeder-feher-zaehlt.de). A team of experts analyse them later and the exemplary reports are published. Even an opportunity for users to comment is provided in this system. This system has found good acceptance among the readers since its inception and the anonymous reporting is steadily growing. It is to be seen how such systems are going to help us take preventive steps and minimise the medication errors.

World Health Organisation (WHO) has already addressed the issue and has created increased awareness by intense campaigning, not only amongst the health care professionals but also amongst the general population. There are currently two Global Patient Safety Challenges advocated by WHO. The first one is “the sustainable hand hygiene improvement” and the second one is “safe surgery, save lives” The goal of the second challenge is to improve the safety of surgical care in all healthcare settings. The WHO Surgical Safety Checklist improves compliance with standards and decreases the incidence of complications. WHO has even promoted and funded the research on patient safety and has made available several resources for the professionals to follow, educate and train.[9] It has taken note of the medication errors, especially the dispensing errors.[10] WHO is in the process of developing a standardised Global Patient Safety Taxonomy for this purpose of dispensing errors with the hope that it will help in development of error reduction strategies.

It is time we think, deliberate and discuss about establishing such critical Incident Reporting and learning system in India, keeping in mind the circumstances under which the anaesthesiologists work in this subcontinent. Black or white, pearls are always precious.
